# Multi-ancestry epigenome-wide analyses identify methylated sites associated with aortic augmentation index in TOPMed MESA

**DOI:** 10.1038/s41598-023-44806-z

**Published:** 2023-10-17

**Authors:** Xiaowei Hu, Jeongok G. Logan, Younghoon Kwon, Joao A. C. Lima, David R. Jacobs, Daniel Duprez, Lyndia Brumback, Kent D. Taylor, Peter Durda, W. Craig Johnson, Elaine Cornell, Xiuqing Guo, Yongmei Liu, Russell P. Tracy, Thomas W. Blackwell, George Papanicolaou, Gary F. Mitchell, Stephen S. Rich, Jerome I. Rotter, David J. Van Den Berg, Julio A. Chirinos, Timothy M. Hughes, Francine E. Garrett-Bakelman, Ani Manichaikul

**Affiliations:** 1 Center for Public Health Genomics, University of Virginia, Charlottesville, VA 22908 USA; 2 School of Nursing, University of Virginia, Charlottesville, VA USA; 3 Department of Medicine, University of Washington, Seattle, WA USA; 4 Department of Internal Medicine, School of Medicine, Johns Hopkins University, Baltimore, MD USA; 5 Division of Epidemiology, School of Public Health, University of Minnesota, Minneapolis, MN USA; 6 Cardiovascular Division, University of Minnesota, Minneapolis, MN USA; 7 Department of Biostatistics, University of Washington, Seattle, WA USA; 8 The Institute for Translational Genomics and Population Sciences, Department of Pediatrics, The Lundquist Institute for Biomedical Innovation at Harbor-UCLA Medical Center, Torrance, CA USA; 9 Laboratory for Clinical Biochemistry Research, University of Vermont, Burlington, VT USA; 10 Division of Cardiology, Department of Medicine, Duke University, Durham, NC USA; 11 Department of Biostatistics, School of Public Health, University of Michigan, Ann Arbor, MI USA; 12 Epidemiology Branch, National Heart, Lung and Blood Institute, Bethesda, MD USA; 13 Cardiovascular Engineering, Inc, Norwood, MA USA; 14 Department of Preventive Medicine and Center for Genetic Epidemiology, Keck School of Medicine, University of Southern California, Los Angeles, CA USA; 15 Division of Cardiovascular Medicine, Department of Medicine, Perelman School of Medicine, University of Pennsylvania, Philadelphia, PA USA; 16 Department of Internal Medicine - Section of Gerontology and Geriatric Medicine, and Department of Epidemiology and Prevention, Wake Forest School of Medicine, Winston-Salem, NC USA; 17 Department of Biochemistry and Molecular Genetics, Department of Medicine, University of Virginia, 1340 Jefferson Park Ave., Pinn hall 6054, Charlottesville, VA 22908 USA

**Keywords:** Epigenomics, DNA methylation, Cardiovascular biology

## Abstract

Despite the prognostic value of arterial stiffness (AS) and pulsatile hemodynamics (PH) for cardiovascular morbidity and mortality, epigenetic modifications that contribute to AS/PH remain unknown. To gain a better understanding of the link between epigenetics (DNA methylation) and AS/PH, we examined the relationship of eight measures of AS/PH with CpG sites and co-methylated regions using multi-ancestry participants from Trans-Omics for Precision Medicine (TOPMed) Multi-Ethnic Study of Atherosclerosis (MESA) with sample sizes ranging from 438 to 874. Epigenome-wide association analysis identified one genome-wide significant CpG (cg20711926-*CYP1B1*) associated with aortic augmentation index (AIx). Follow-up analyses, including gene set enrichment analysis, expression quantitative trait methylation analysis, and functional enrichment analysis on differentially methylated positions and regions, further prioritized three CpGs and their annotated genes (cg23800023-*ETS1*, cg08426368-*TGFB3*, and cg17350632-*HLA-DPB1*) for AIx. Among these, *ETS1* and *TGFB3* have been previously prioritized as candidate genes. Furthermore, both *ETS1* and *HLA*-*DPB1* have significant tissue correlations between Whole Blood and Aorta in GTEx, which suggests *ETS1* and *HLA*-*DPB1* could be potential biomarkers in understanding pathophysiology of AS/PH. Overall, our findings support the possible role of epigenetic regulation via DNA methylation of specific genes associated with AIx as well as identifying potential targets for regulation of AS/PH.

## Introduction

Arterial stiffness (AS) serves as an independent predictor of cardiovascular diseases (CVD) morbidity and mortality^1,2^. Structurally, AS is characterized by degeneration of the media layer of arterial walls and involves decreased intact elastin and increased collagen fibers^3^. Functionally, AS impairs the cushioning capacity of arteries that transform pulsatile flow at the ascending aorta into steady flow through the arterioles^3^. Pulsatile hemodynamics (PH) resulting from AS involves the augmentation of systolic blood pressure (BP) in late systole^4^ which partially results from wave reflection arriving at the aorta during ejection, increasing left ventricular load^5^.

Both genome-wide association studies (GWAS) and candidate gene approaches have identified genetic variants that are associated with AS/PH, including those in the renin–angiotensin–aldosterone (RAA) system, elastic fiber structural components, metalloproteinases, the nitric oxide (NO) pathway, and β-adrenergic receptors^6–8^. However, a large group of identified AS/PH-associated genes are not related to known pathophysiological mechanisms of AS/PH, reflecting the likely multifactorial etiology of AS/PH^6^. Since variation in AS/PH is mediated through gene expression, exploration of factors that are involved in transcriptional pathways may offer new insights to variation of AS/PH.

DNA methylation (DNAm), one of the most studied epigenetic modifications, plays a critical role in the transcriptional regulation^9,10^. DNAm occurs when a methyl group is added to a cytosine followed by guanine (CpG) in the genome^10^. Despite the emphasis on the role of AS/PH in the development of CVD, few human studies have examined DNAm in relation to AS/PH. In contrast, studies in aged mice have demonstrated that compound H, a potential activator of DNA demethylases, attenuates aging-related AS and hypertension^11^. In hyperhomocysteinemia mice, 5-aza-2’-deoxycytidine (Aza), a DNA methyltransferase (*DNMT1*) inhibitor, reduced high BP and vascular stiffening via reduced expression of matrix metalloproteinases 9 (*MMP9*), and tissue inhibitors of metalloproteinases (TIMPs)^12^. Using umbilical cord blood DNA from 470 participants, an epigenome-wide association study (EWAS) identified differentially methylated CpGs associated with increased BP and AS at an 8–9-year follow-up examination^13^. Although single CpG positions have been associated with many phenotypes^14–16^, various studies have reported that methylation levels are strongly correlated across the genome, and the reported functionally-relevant findings are generally associated with genomic region rather than single CpG position^17–19^. Hence, understanding DNAm associations with AS/PH at both single CpG position and genomic region levels are important to gain a broader understanding of DNAm changes in AS/PH.

Our study aimed to identify differentially methylated CpG positions and regions that are associated with AS/PH traits. First, we examined differently methylated positions (DMPs) and differentially methylated regions (DMRs) that are associated with eight AS/PH traits, using both epigenome-wide and literature-based candidate gene approaches^7^. Second, we conducted three follow-up analyses including gene set enrichment analysis, expression quantitative trait methylation analysis, and functional enrichment analysis for significantly associated DMPs and DMRs for each AS/PH trait. Third, we prioritized CpGs and their annotated genes for each AS/PH trait using the overlap of significant findings across three follow-up analyses.

## Results

**Figure 1:**
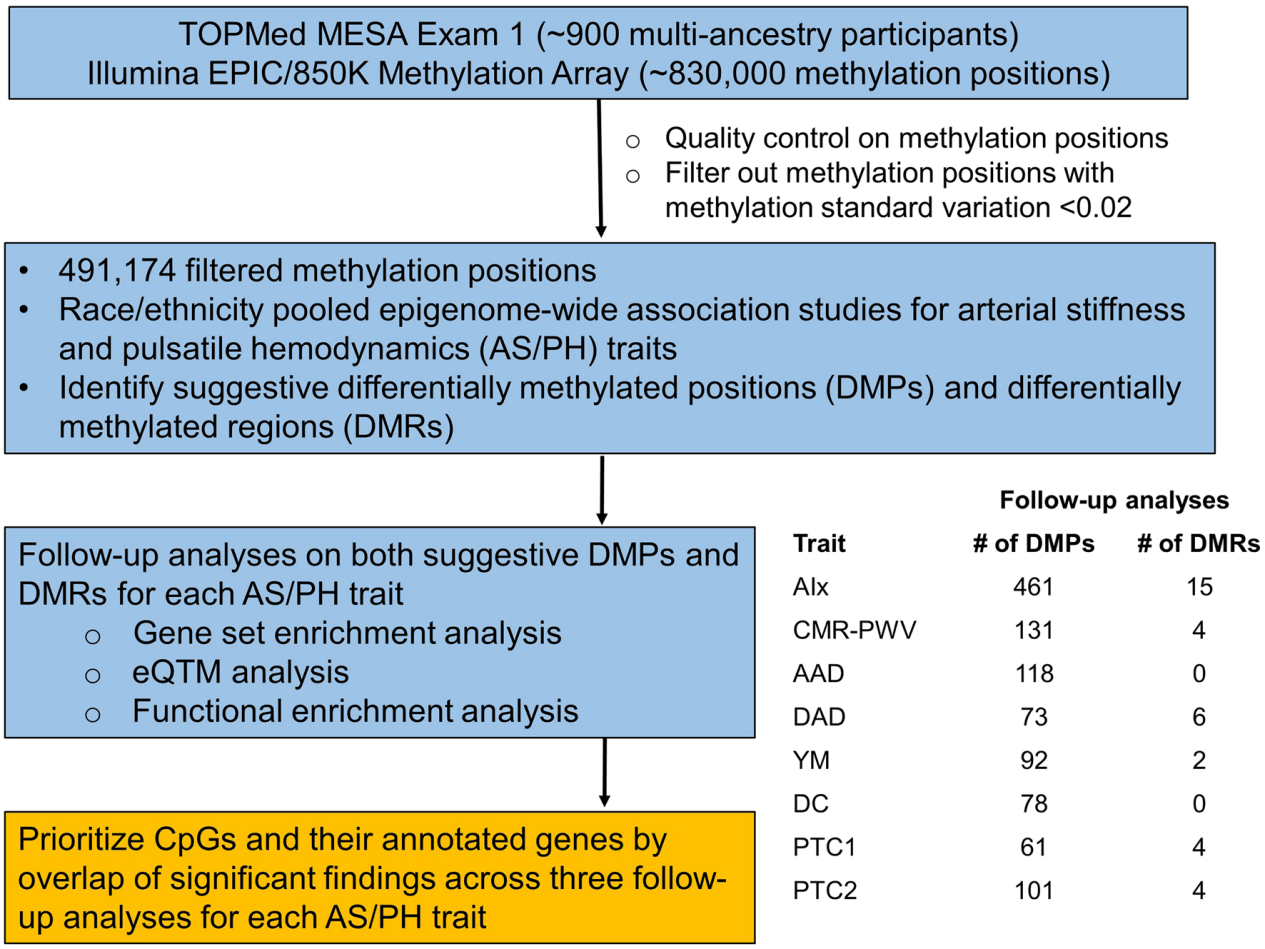
Study design. The suggestive DMP was defined as CpG with association p-value less than 1 × 10^−4^ and 0.05 respectively for epigenome-wide and candidate-gene approaches; the suggestive DMR was defined as co-methylated region with association p-value less than 5 × 10^−4^ and 0.05 respectively for epigenome-wide and candidate-gene approaches; eQTM, expression quantitative trait methylation; AIx, aortic augmentation index; CMR-PWV, aortic arch pulse-wave velocity measured by cardia magnetic resonance imaging; AAD, ascending aortic distensibility; DAD, descending aortic distensibility; YM, Young’s Elastic Modulus; DC, distensibility coefficient; PTC1 and PTC2, radial artery pressure waveform index 1 and 2.

The overview of study design is shown in Fig. 1. After identifying suggestive differentially methylated positions (DMPs) and differentially methylated regions (DMRs) through race/ethnicity pooled epigenome-wide association studies, we performed three follow-up analyses on both suggestive DMPs and DMRs to prioritize CpGs and their annotated genes for each AS/PH trait. The assessment of Alx yielded prioritized genes from the subsequent three follow-up analyses.

### Participant characteristics

The demographic and clinical characteristics of study samples for AIx are summarized in Table 1, which includes 805 TOPMed MESA participants who have both AIx and DNAm measures from MESA Exam 1 (Methods). Based on participant self-reported race/ethnicity, 41% and 33% of the participants were categorized as Non-Hispanic White (NHW) and Hispanic/Latino (HIS) respectively. The remaining participants represented African American (AA) (18%) and Chinese (8%). The median value of AIx was 14.47 (25th percentile = 13.54 and 75th percentile = 15.83). After conducting linear regression to examine the association of log-transformed AIx with each clinical characteristic, we found that age, sex, AA group, mean arterial pressure, anti-hypertensive medication, and HDL cholesterol had nominally significant associations (*P* < 0.05). The sample characteristics, and association of the other seven AS/PH traits with each clinical characteristic are summarized in Supplementary Table S1.

**Table 1:** Clinical characteristics of samples in TOPMed MESA.

	N = 805		
	Summary statistics	Estimate (se)	*P*-value
Age, year	60 ± 10	0.0037 (0.0004)	6.89E-16
Male (%)	48	− 0.0869 (0.0086)	1.04E-22
Non-Hispanic White (%)	41	Baseline	Baseline
African American (%)	18	0.0264 (0.0128)	0.04
Hispanic/Latino (%)	33	0.0087 (0.0107)	0.41
Chinese (%)	8	− 0.0217 (0.0178)	0.22
BMI (kg/m^2^)	28.80 ± 5.23	− 0.0007 (0.0009)	0.44
Never smoker (%)	49	Baseline	Baseline
Former smoker (%)	37	− 0.0161 (0.0099)	0.10
Current smoker (%)	13	− 0.0193(0.0142)	0.18
Smoking pack-years	9.68 ± 17.65	− 0.0002 (0.0003)	0.49
Mean arterial pressure, mmHg	88.86 ± 12.12	0.0014 (0.0004)	2.42E-04
Anti-hypertensive medication (%)	34	0.0309 (0.0096)	1.29E-03
Anti-diabetic medication (%)	8	− 0.0153 (0.0173)	0.37
Lipid-lowering medication (%)	16	− 0.0049 (0.0124)	0.69
Fasting glucose (mg/dL)	95.24 ± 24.16	− 0.0002 (0.0002)	0.30
HDL cholesterol (mg/dL)	50.52 ± 13.96	0.0015 (0.0003)	2.33E-06
LDL cholesterol (mg/dL)	118.13 ± 31.57	2.54E-05 (0.0001)	0.86
Triglycerides (mg/dL)	135.24 ± 79.88	− 3.28E-05 (5.72E-05)	0.57
AIx (%)	14.47 (13.54, 15.83)		

Mean ± sd; median (25th percentile, 75th percentile); estimate (se) and *P*-value, estimated effect size (standard error) and *P*-value from linear regression model between log-transformed aortic augmentation index (AIx) and each clinical characteristic.

### Two FDR-significant CpGs associated with AIx

**Figure 2:**
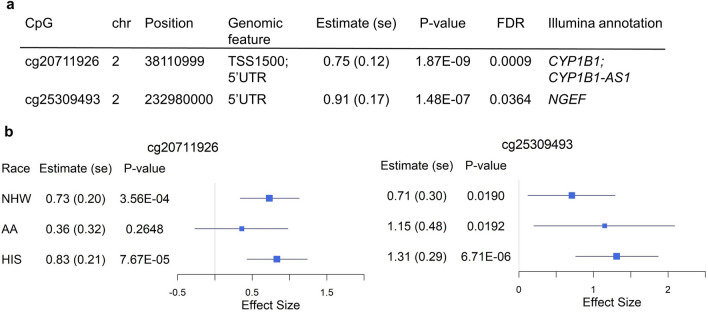
FDR-significant (FDR < 0.05) differentially methylated positions associated with aortic augmentation index. (**a**) Multi-ancestry epigenome-wide association studies (EWAS) summary statistics of two FDR-significant CpGs. (**b**) Forest plots of race-stratified EWAS of two FDR-significant CpGs. Position, based on GRCh38/hg38; Estimate (se) and *P*-value, effect size (standard error) and *P*-value of CpG respectively from EWAS; FDR, false discovery rate based on multiple testing correction on 491,174 CpGs; NHW, Non-Hispanic White; AA, African American; HIS, Hispanic/Latino.

After applying standard methylation quality control and filtering out positions with low methylation variation (Methods), there were 491,174 CpGs identified for epigenetic association analyses in MESA. In the multi-ancestry epigenome-wide association studies (EWAS) of AS/PH traits, we first reported the Quantile–Quantile (QQ) plots with corresponding λ, a measure to quantify the inflation in the test statistics (Supplementary Fig. S1). The genomic inflation λ ranged from 0.97 (PTC1) to 1.05 (AIx). We observed two false discovery rate (FDR)-significant (FDR < 0.05) differentially methylated positions (DMPs), cg20711926 (*CYP1B1*) and cg25309493 (*NGEF*), associated with AIx, with cg20711926 (*p*-value = 1.87 × 10^−9^) also passing Bonferroni significance (0.05/491,174 = 1.02 × 10^−7^) (Fig. 2). We further examined the association of these two FDR-significant DMPs with AIx in race/ethnic stratified analyses. The two DMPs were nominally significant in all ancestry groups except cg20711926 in AA group (Fig. 2), with greater statistical significance in HIS than for the other two ancestry groups. Additionally, two FDR-significant DMPs were positively associated with AIx in both race/ethnic pooled and stratified analyses. However, there were no FDR-significant DMPs for the other seven AS/PH traits as shown in Supplementary Table S2. In analysis of CpGs annotated to genes in the candidate-gene list (Supplementary Table S3), there were no CpGs identified at FDR < 5% (Supplementary Table S2).

As there were few FDR-significant DMPs identified, we interrogated those CpGs that achieved suggestive significance based upon EWAS (*p*-value < 1 × 10^−4^) and candidate gene (*p*-value < 0.05) approaches to select CpGs for follow-up analyses. The suggestive thresholds for statistical significance were selected based on the distribution of EWAS p-values of all eight AS/PH traits (Supplementary Tables S4-S11). Accordingly, there were 461 unique suggestive DMPs included in the follow-up analyses for AIx (Supplementary Table S4). The suggestive DMPs included in the follow-up analyses for the other seven AS/PH traits are presented in Supplementary Tables S5-S11.

### EWAS at co-methylated genomic regions

We tested 58,222 pre-defined genomic regions and identified 8,165 co-methylated genomic regions for the identification of differentially methylated regions (DMRs) associated with AS/PH traits. There was no FDR-significant DMR identified by either the epigenome-wide or candidate-gene approach for any of the AS/PH traits (Supplementary Table S12). Similar to suggestive DMPs from EWAS, we reported and used co-methylated genomic regions reaching suggestive significance of association p-values from two approaches (p-value < 5 × 10^−4^ from epigenome-wide approach and p-value < 0.05 from candidate-gene approach) for follow-up analyses.

Overall, we identified suggestive DMRs for six out of eight AS/PH traits (no suggestive DMR for traits AAD and DC, Supplementary Table S12). The association results of suggestive DMRs and their inclusive CpGs for six AS/PH traits are presented in Supplementary Tables S13-S18. AIx had the most suggestive DMRs where 13 and 2 DMRs were from epigenome-wide and candidate-gene approaches, respectively (Table 2). Among these, 13 out of 15 suggestive DMRs were positively associated with AIx. Overall, the number of CpGs included in suggestive DMRs ranged from 3 to 7. Further, we checked the overlap between suggestive DMPs and suggestive DMRs. Supplementary Table S19 shows that methylation differences at individual CpGs and co-methylated regions partially overlap, demonstrating the value of analyzing both individual CpGs and co-methylated regions provided a more complete view of DNAm association with AS/PH traits.

**Table 2:** Suggestive differentially methylated regions associated with aortic augmentation index.

Analysis approach	Suggestive significance of *P*-value	DMR (GRCh38/hg38)	No. CpGs	Estimate (se)	*P*-value	Illumina annotation
Epigenome-wide	5 × 10^−4^	chr11:2,300,571–2,300,821	7	0.72 (0.18)	9.05E-05	*C11orf21;TSPAN32*
		chr14:22,819,785–22,819,906	3	0.51 (0.13)	1.50E-04	*SLC7A7*
		chr22:44,676,732–44,677,170	4	0.49 (0.13)	1.61E-04	*PRR5*
		chr4:7,646,814–7,646,901	3	0.73 (0.19)	1.86E-04	*SORCS2*
		chr1:153,775,067–153,775,183	5	0.43 (0.12)	2.07E-04	*SLC27A3*
		chr13:40,188,014–40,188,333	6	− 0.62 (0.17)	3.32E-04	*LINC00332*
		chr13:25,095,914–25,096,182	7	1.21 (0.34)	3.36E-04	*PABPC3*
		chr19:48,720,589–48,720,909	3	1.18 (0.33)	3.75E-04	*MAMSTR; RASIP1*
		chr17:35,241,580–35,241,948	3	− 0.57 (0.16)	3.88E-04	*SLFN5*
		chr12:6,533,036–6,533,189	3	0.55 (0.16)	4.07E-04	*GAPDH*
		chr17:30,970,363–30,970,587	6	1.61 (0.46)	4.49E-04	*RNF135*
		chr8:133,576,001–133,576,067	3	1.02 (0.29)	4.82E-04	N/A
		chr9:35,905,820–35,906,152	4	0.50 (0.14)	4.84E-04	*HRCT1*
Candidate-gene	0.05	chr20:46,008,193–46,008,343	3	0.40 (0.15)	6.42E-03	*MMP9*
		chr7:74,062,328–74,062,346	3	0.28 (0.13)	0.0298	*ELN*

DMR, differentially methylated region; Estimate (se) and *P*-value, effect size (standard error) and *P*-value of co-methylated regions respectively from co-methylated region association test by *coMethDMR*; No. CpGs, number of CpGs included in the DMR.

### Gene set enrichment analysis

We conducted gene set enrichment analysis (Gene Ontology (GO) and Kyoto Encyclopedia of Genes and Genomes (KEGG) pathway analysis) for two sets of CpGs, the significant EWAS set and the significant WGCNA-module set, to identify FDR-significant (FDR < 0.05) GO terms and KEGG pathways for each trait of interest (Methods). By using 518 CpGs that were included in the significant EWAS set associated with AIx, we identified 22 and 36 FDR-significant GO terms and KEGG pathways respectively. The summary and details of FDR-significant enrichment results using CpGs included in the significant EWAS set for AS/PH traits is presented in Supplementary Table S20 and Supplementary Tables S21-S28 correspondingly. From WGCNA analysis, we identified five AIx-associated modules with nominal significance (*p* < 0.05), while no significant modules were identified for PTC1 and PTC2 (Supplementary Table S29). The turquoise module had the most significant enrichment results for AIx, 448 and 48 FDR-significant GO terms and KEGG pathways were identified respectively (Supplementary Table S29). The two FDR-significant CpG sites for AIx, cg20711926 (*CYP1B1*) and cg25309493 (*NGEF*), were also included in the turquoise module. The summary of FDR-significant enrichment results using CpGs included in the significant WGCNA-module set for AS/PH traits is presented in Supplementary Table S29.

We further examined the overlap of significant enrichment results from the two sets of CpGs. There were seven overlapping significant GO terms and eleven overlapping significant KEGG pathways from the turquoise module for AIx, while there was only one overlapping significant GO term from the purple module (Supplementary Table S29). The top overlapping significant GO term for AIx from the turquoise module was a biological process regulating systemic arterial BP (GO:0,001,990), and the only overlapping significant GO term from the purple module is also a biological process regulating inflammatory response (GO:0,050,727) (Supplementary Table S30). Furthermore, 13 candidate genes (*ACE, AGER, AGTR1, ECE1, EDNRB, ESR1, ETS1, IL6, MMP9, NFKB1, NOS3, REN, TNF*) were involved in these two overlapping significant GO terms for AIx (Supplementary Table S30). For the other seven AS/PH traits, the overlap of significant enrichment results was only observed for AAD and DC (Supplementary Tables S29, S31, S32).

### Significant associations between gene expression and suggestive DNAm differences

To identify genes whose expression is associated with suggestive DNAm differences, we conducted expression quantitative trait methylation (eQTM) analysis for suggestive DMPs and DMRs with their annotated genes (Methods). We identified 60 FDR-significant eQTMs at DMPs among 343 expression-DMP association tests for AIx (Supplementary Table S33). Fewer FDR-significant eQTMs at DMPs were observed for the other seven AS/PH traits (range from 2 to 13, Supplementary Table S34). Additionally, there were five FDR-significant eQTMs at DMRs for AIx, and no more than two significant eQTMs at DMRs identified for each of DAD, YM, PTC1, and PTC2 (Supplementary Table S35).

### Significant enrichment in Heart enhancers for AIx-associated DNAm differences

We conducted functional enrichment analysis to better understand the complex interpretation of suggestive DNAm differences (Methods). Specifically, we checked the enrichment results of two AS/PH-relevant tissues (Blood and Heart) for suggestive DMPs and DMRs. The analysis for AIx highlighted enrichment in both Blood and Heart enhancers from 15 chromatin states for CpGs included in AIx-associated DMPs and DMRs (Supplementary Fig. S2). Furthermore, the significant enrichment in Heart enhancers was observed in two AS/PH-relevant cell-types: E104 Right Atrium (Q-value = 8.6 × 10^−3^) and E65 Aorta (Q-value = 0.04) (Supplementary Table S36). For the other seven AS/PH traits, we did not observe any significant enrichment in the 15 chromatin states for either Blood or Heart tissues (Supplementary Fig. S3-S9).

### Three genes prioritized for AIx

**Figure 3:**
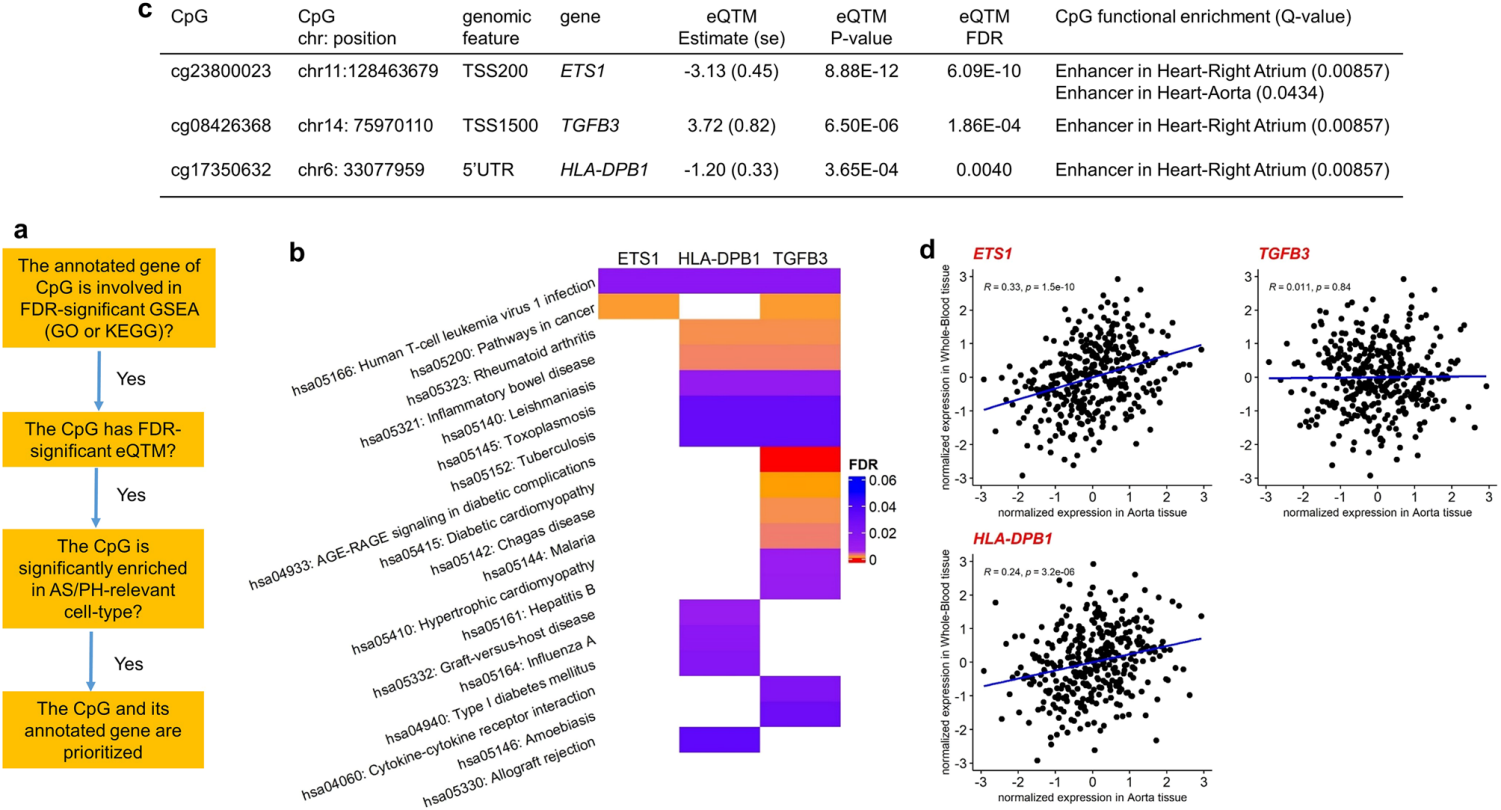
Three prioritized genes (*ETS1*, *HLA-DPB1*, *TGFB3*) for aortic augmentation index. (**a**) Workflow of prioritization; (**b**) FDR-significant KEGG pathways that three prioritized genes were involved; (**c**) Summary table of follow-up analyses results of three prioritized CpGs associated with aortic augmentation index; (**d**) Pearson’s correlation (R) of inverse-normalized gene expressions between GTEx v8 Whole-Blood and Aorta tissues for three prioritized genes. Position, based on (GRCh38/hg38); FDR, false discovery rate; functional enrichment, eFORGE 15 chromatin state enrichment analysis; GSEA, gene set enrichment analysis; GO, Gene Ontology; KEGG, Kyoto Encyclopedia of Genes and Genomes; eQTM, expression quantitative trait methylation; AS/PH, arterial stiffness and pulsatile hemodynamics.

After conducting follow-up analyses for CpGs included in suggestive DMPs and DMRs, we checked CpGs included in the overlap of significant findings across three follow-up analyses (gene set enrichment analysis, expression quantitative trait methylation analysis, and functional enrichment analysis). The work flow of prioritization is shown in Fig. 3a. We first summarized results from the three follow-up analyses for the two FDR-significant DMPs associated with AIx in Supplementary Table S37. No significant results were observed in any of the three follow-up analyses for these two DMPs. However, there were three other CpGs (cg23800023-*ETS1,* cg17350632-*HLA-DPB1, and* cg08426368-*TGFB3*) prioritized by follow-up analyses for AIx.

Among the prioritized genes, both *ETS1* and *TGFB3* are on our candidate gene list, although the candidate gene list itself was not used in our overall approach for prioritization of genes. All three genes were involved in KEGG pathway hsa05166: Human T-cell leukemia virus 1 infection pathway and both *ETS1* and *TGFB3* were in KEGG pathway hsa05200: Pathways in cancer (Fig. 3b). Additionally, all three CpGs were FDR-significant eQTMs and both CpGs cg23800023 and cg17350632 were negatively associated with their annotated genes, *ETS1* and *HLA-DPB1*, respectively (Fig. 3c). Furthermore, the CpG cg23800023 was enhancer-enriched in both AS/PH-relevant cell-types, Right Atrium (enrichment Q-value = 8.57 × 10^−3^) and Aorta (enrichment Q-value = 0.0434); the other two CpGs were enhancer-enriched in Right Atrium (enrichment Q-value = 8.57 × 10^−3^) (Supplementary Fig. S2 and Fig. 3c). Finally, we checked the correlation of gene expression levels between GTEx Whole-Blood and Aorta tissues respectively for these three genes (Methods). Both *ETS1* and *HLA-DPB1* have significant tissue correlation (*ETS1*, Pearson’s correlation R = 0.33 with p-value = 1.50 × 10^−10^; *HLA-DPB1*, Pearson’s correlation R = 0.24 with p-value = 3.20 × 10^−6^; Fig. 3d). However, *TGFB3* was not significantly correlated in those two tissues in GTEx. We also checked the overlap of significant findings across the three follow-up analyses for the other seven AS/PH traits, but no overlap was observed.

## Discussion

Arterial stiffness (AS) is a subclinical condition that has a significant prognostic value for future development of CVD events and end organ damage^20^. AS results in excess pulsatile hemodynamics (PH), which can be measured by aortic augmentation index^21^ (AIx). While CVD is often identified late requiring interventions to manage disease progress, targeting aortic AS and excessive PH present an early opportunity to identify individuals at greater risk as well as means to monitor effects of preventative interventions. Although the pathophysiology and prognostic significance of AS/PH have been well described, epigenetic influences that impact transcript level expression of AS/PH remain poorly known. The current study contributes to the field by investigating individual CpG positions and co-methylated regions that are associated with multiple AS/PH traits using both epigenome-wide and candidate-gene approaches. Among a diverse cohort of individuals free from clinical CVD, we identified two significant differentially methylated positions (DMPs), cg20711926 (Bonferroni-significant) and cg25309493 (FDR-significant) that were associated with AIx independent of potential confounders. These two DMPs were positively associated with AIx across all groups in race/ethnic stratified analysis.

AIx is a commonly used measure of late systolic pressure augmentation obtained from pulse wave analysis (PWA)^22^. PWA is a noninvasive method to generate the ascending aorta pressure wave from the pressure waveform measured in the radial artery^21^. The pressure waveform is a composite of the forward pressure wave (incident wave) and a reflected wave. In elastic vessels, because pulse wave velocity (PWV) is low, the reflected wave tends to arrive back at the aortic root during diastole. In stiff vessels, PWV is high and the reflected wave arrives back at the central arteries earlier, augmenting the systolic pressure. This augmented pressure (AP) is calibrated by PWA, and AIx is defined as AP which is expressed as a percentage of pulse pressure^23^. While PWV is the gold standard of AS^24^, AIx can be affected by multiple factors (e.g., left ventricular ejection, PWV, timing of reflection, arterial tone, structure at peripheral reflecting sites, BP, and heart rate)^25^. Nevertheless, the current study demonstrated that AIx was the only AS/PH trait that identified the most significant associated DNAm differences among eight AS/PH traits, including CMR-PWV, AAD, DAD along with all other derives of PWA including YM, DC, PTC 1 & 2.

*CYP1B1* (cytochrome P450 family 1 subfamily B member 1) is the gene annotated to cg20711926, the Bonferroni-significant DMP associated with AIx. *CYP1B1* is a member of the CYP1 subfamily and encodes *CYP1B1* enzyme which is involved in drug metabolism and synthesis of cholesterol, steroids, and other lipids^26^. While many studies reported the role of *CYP1B1* in glaucoma and cancer^27,28^, recent studies have indicated that *CYP1B1* is involved in cardiac pathophysiological changes. For example, *CYP1B1* has been found to mediate angiotensin II–induced aortic smooth muscle cell migration, proliferation, and protein synthesis in rats^29^, as well as to contribute to cardiac hypertrophy induced by uremic toxin in mice^30^. Our finding further suggests that methylation of *CYP1B1* may play a crucial role in determining AIx, although additional research is required to determine whether the association we observed is causal. Despite the need for further investigation, *CYP1B1* may have noteworthy therapeutic implications for cardiac hypertrophy and AS/PH that are the important underlying mechanisms of CVD^31^.

In addition to *CYP1B1*, our study also prioritized three CpGs and their annotated genes (cg23800023-*ETS1*, cg08426368-*TGFB3*, and cg17350632-*HLA-DPB1*) for AIx, based on the overlap of significant results from three follow-up analyses of DMPs, including gene set enrichment analysis, expression quantitative trait methylation (eQTM) analysis, and functional enrichment analysis. Both *ETS1* and *TGFB3* have been previously prioritized as candidate genes^7^, although the candidate gene information was not used in our overall approach for the prioritization. Thus, our approach brings new independent evidence to support the roles of these two genes in processes regulating AS/PH.

First, the cg23800023 annotated gene *ETS1* encodes the founding member of the family of ETS transcription factors. ETS family proteins have a conserved ETS DNA-binding domain that recognizes the GGAA/T in target genes and function as transcriptional activators or repressors of many genes^32^. In endothelial cells, vascular smooth muscle cells, and epithelial cancer cells, ETS is involved in regulating expressions of matrix metalloproteinase (MMP)1, 2, 3, and 9 and vascular endothelial growth factor^33–36^ that are the well-known regulators of AS/PH^37^. Our study adds to the existing evidence supporting the link between *ETS1* and AIx via the regulation of DNAm. However, further research is needed to establish the causality of this association.

Second, the cg17350632 annotated gene *HLA-DPB1* (histocompatibility complex, class II, DP beta 1) belongs to a family of human leukocyte antigen (HLA) complex genes that help the immune system to distinguish the body’s own proteins from proteins from foreign bodies. Among three subregions (DP, DQ, and DR) of HLA-D, HLA-DP molecule has not been extensively studied^38^, but available studies have demonstrated that HLA-DPB1 is associated with autoimmune disorder such as rheumatoid arthritis^39^ and Behcet’s disease^40^. The role of *HLA-DPB1* in the vascular system has not been revealed yet. Our study presents novel information on the connection between *HLA-DPB1* and AIx via the regulation of DNAm. Furthermore, both *ETS1* and *HLA-DPB1* have significant tissue correlations between Whole Blood and Aorta in GTEx, which suggests *ETS1* and *HLA*-*DPB1* could be potential biomarkers in understanding pathophysiology of AS/PH.

Lastly, the cg08426368 annotated gene *TGFB3* (transforming growth factor beta 3) encodes a *TGFB3* protein which is part of a large family of cytokines called TGFB superfamily^41^. *TGFB3* regulates molecules involved in cell proliferation, cell differentiation, and apoptosis, and plays a critical role in the formation of blood vessels and wound healing^41,42^. Previous studies have indicated that TGFB pathways regulate the expressions of elastin, collagen, and *MMP2* &*9*^43^, that are the major determinants of mechanical properties of large arteries^44^. Although *TGFB3* did not show a significant tissue correlation between Whole Blood and Aorta in GTEx, the present study further confirms the potential relationship between *TGFB3* and AIx mediated by DNAm.

It is noteworthy that all these three prioritized genes were involved in KEGG pathway hsa05166, Human T-cell leukemia virus 1 (HTLV-1) infection pathway. HTLV-1 infection can cause adult T-cell leukemia/lymphoma and HTLV-1- associated myelopathy^45,46^, as well as an inflammatory disease such as arthritis^47^. HTLV-1 is known to disturb the regulation of cytokines including interferon gamma (IFN-γ), tumor necrosis factor alpha (TNF-α), transforming growth factor beta (TGF-β), and IL-10^48^. Given the previous evidence that HTLV-1 triggers chronic inflammatory cascade, which is an important risk factor of atherosclerosis and CVD^49^, potential roles of HTLV infection in AS/PH are considered as an important gap that needs to be filled by future studies.

In summary, our study provides valuable information on epigenetic modifications associated with AS/PH. The major strengths of our study include being one of the very few studies that examined the epigenome-wide association with AS/PH and the use of population-based multi-ancestry data that include surrogate measures of AS and related hemodynamic parameters. In addition, we conducted a series of follow-up analyses to prioritize genes that are potentially relevant to AS/PH. A few limitations of our study should be noted. First, the study didn’t use AS gold standard measure, carotid–femoral PWV^50^, due to data unavailability. Secondly, our study has limited statistical power of identifying significant DNAm differences associated with AS/PH traits due to the sample size. Thirdly, our study lacks a replication study due to the discrepancies in AS/PH measures across public data. Lastly, the study sample included relatively old adults with mean age of 60 (± 10) years; thus, the study findings may be confounded with other CVD risks associated with aging. Future studies should aim to replicate the study findings in younger and healthy populations and to investigate the biological processes in which the identified genes contribute to AS/PH. Such studies will help better understand pathophysiology of AS/PH and identify potential therapeutic targets of AS/PH.

## Methods

### Overview of approach

The overview of study design is shown in Fig. 1. We conducted analyses using Illumina’s Methylation EPIC BeadChip (850 K) and NHLBI Trans-Omics for Precision Medicine (TOPMed) Multi-Ethnic Study of Atherosclerosis (MESA) Exam 1 arterial stiffness and pulsatile hemodynamics (AS/PH) traits with about 900 multi-ancestry participants. We first applied quality control (QC) on DNA methylation (DNAm) data and filtered out methylation positions with low methylation variation (i.e., the standard deviation of methylation Beta-values < 0.02). The epigenetic analyses were then performed on the filtered methylation positions. First of all, we performed epigenome-wide association studies (EWAS) between AS/PH traits and DNAm at individual CpG position and co-methylated genomic region in race/ethnicity pooled analyses respectively. Then the CpGs included in both differentially methylated positions (DMPs) and differentially methylated regions (DMRs) were examined further in three follow-up analyses (i.e., gene set enrichment analysis, expression quantitative trait methylation analysis, and functional enrichment analysis) for each AS/PH trait. Finally, we used the overlap of significant findings across three follow-up analyses to prioritize CpGs and their annotated genes for each AS/PH trait.

### Study participants

The study population consisted of participants free from clinical CVD enrolled in MESA, a longitudinal study of subclinical CVD and risk factors that predict progression to clinically overt cardiovascular disease or progression of the subclinical disease^51^. Between 2000 and 2002, MESA recruited 6,814 men and women 45 to 84 years of age from Forsyth County, North Carolina; New York City; Baltimore; St. Paul, Minnesota; Chicago; and Los Angeles. Exclusion criteria were clinical CVD, weight exceeding 136 kg (300 lb.), pregnancy, and impediment to long-term participation. Approximately 38 percent of the recruited participants are white, 28 percent African American, 22 percent Hispanic/Latino, and 12 percent Asian, predominantly of Chinese descent. Among the specific exclusions related to CVD were the following physician-diagnosed criteria: heart attack, angina or taking nitroglycerin, stroke or TIA, heart failure, current atrial fibrillation, or having undergone procedures related to cardiovascular disease (https://www.mesa-nhlbi.org/eligibility.aspx). All research was performed in accordance with relevant guidelines and regulations. All participants provided informed consent and the protocols of MESA were approved by the IRBs of collaborating institutions and the National Heart, Lung and Blood Institute and informed consent was obtained from all participants. Research involving human research participants was performed in accordance with the Declaration of Helsinki. The participants in this study are a subset of MESA individuals who have both AS/PH measures (described in detail previously^52–56^) and DNAm measures^57^ from MESA Exam 1 (2000–2002).

### DNA methylation profiling

DNAm profiles were generated using the Infinium Methylation EPIC BeadChip (850 K) (Illumina, Inc., San Diego, CA). As part of the TOPMed MESA Multi-Omics project, 900 participants were selected for MESA Exam 1 DNAm profiling based on the following criteria: (1) restrict to those already included in the TOPMed Whole Genome Sequencing effort^57^, (2) preserve the race/ethnic distribution of participants in the parent MESA cohort, (3) maximize the amount of overlapping ‘omics data (with the other ‘omics included in the TOPMed MESA Multi-Omics pilot requiring availability of plasma samples for proteomics/metabolomics and RNA from PBMCs, monocytes or T cells for RNA-seq). The QC was applied to DNAm data prior to analysis, including color bias correction, median background adjustment, standard quantile adjustment, batch effect correction (using *ComBat* function in *sva*^58^ R package), sex or race mismatch check, and outlier detection (using *Gaphunter* function in *minifi*^59^ R package). We further filtered out low methylation variation positions that the standard deviation of their methylation Beta-values is less than 0.02. Hence, 491,174 CpGs were left for epigenetic analyses. To preserve better statistical properties (i.e., homoscedasticity), the M-values (i.e., M = log(Beta/(1-Beta))) were used in the analyses.

### Arterial stiffness and pulsatile hemodynamics traits

The study examined eight AS/PH traits, including: aortic augmentation index (AIx, %) measured by PWA; aortic arch pulse-wave velocity measured by cardiac magnetic resonance imaging (CMR-PWV, m/sec), ascending aortic distensibility (AAD, mmHg), descending aortic distensibility (DAD, mmHg); Young’s Elastic Modulus (YM, mmHg) and distensibility coefficient (DC, mmHg), measured by carotid ultrasound; PTC1 (milliseconds) and PTC2 (milliseconds) obtained from radial artery pressure waveforms. The definition and measurement of all eight AS/PH traits in MESA are described in previous studies^21,54,56^. Due to the skewness of raw phenotype data of eight AS/PH traits, the log-transformation was applied to AS/PH traits except for DC where the square-root transformation was applied.

### EWAS at individual CpG

The linear regression adjusted for covariates was performed to test association between each AS/PH trait and M-value of individual CpG at epigenome-wide scale excluding sex chromosomes for 491,174 CpGs. The covariates included age, sex, race/ethnicity, BMI, smoking status (never, former, or current), smoking pack-years, mean arterial pressure, anti-hypertensive medication usage (yes or no), anti-diabetic medication usage (yes or no), lipid-lowering medication usage (yes or no), fasting glucose, high-density lipoprotein (HDL) cholesterol, low-density lipoprotein (LDL) cholesterol, triglycerides, estimated cell type proportions (Lymph, Mono, and Neu), and the first four genomic principal components (PCs) of ancestry. We carried out pooled analyses across self-reported race/ethnic groups for EWAS. The Illumina^60^ was used for CpG annotation. We then generated a list of 59 candidate genes from literature-based approach^7^. The EWAS results for CpGs annotated to 59 candidate genes were referred to as candidate-gene approach results. All association tests were adjusted for multiple comparisons using false discovery rate (FDR) correction (Benjamini-Hochberg) at 5%. Due to the limited statistical power of this study, we considered CpGs with EWAS p-values passed suggestive significance as suggestive DMPs. The suggestive significance cut-offs for epigenome-wide approach and candidate-gene approach were 1 × 10^−4^ and 0.05, respectively. All suggestive DMPs were then used in the follow-up analyses.

### EWAS at co-methylated genomic region

We applied coMethDMR^61^ on 491,174 CpGs to identify DMRs. First, coMethDMR identified co-methylated sub-regions with closely located and co-methylated CpGs. We first extracted clusters of CpGs located closely within genomic regions, i.e., the CpG cluster has at least three CpGs and the maximum separation between any two consecutive CpGs within the cluster is 200 base pairs. This step helps to ensure the sub-regions with similar CpG densities. Then we used the correlation between methylation levels among CpGs (i.e., *rdrop* statistics > 0.4 in coMethDMR) in a sub-region to identify co-methylated CpGs. Next, the median of M-values of CpGs within a co-methylated region was used to test association with AS trait in a random coefficient mixed model in coMethDMR that allowed us to model both variations between CpGs within the region and differential methylation simultaneously. The mixed model was also adjusted for the same covariates as for EWAS at individual CpG. We used the *AnnotateResults* function in coMethDMR to annotate co-methylated regions. The association results for co-methylated regions annotated to 59 candidate genes were referred to as candidate-gene approach results. Similar to the identification of suggestive DMP, the co-methylated region with association p-values passed suggestive significance was defined as suggestive DMR. The suggestive significance cut-offs for DMR were 5 × 10^−4^ and 0.05 for epigenome-wide approach and candidate-gene approach, respectively. All suggestive DMRs were then used in the follow-up analyses as well.

### Weighted gene co-expression network analysis (WGCNA)

WGCNA^62^ is a systems biology method that can be used to find modules (clusters) with highly correlated methylation levels and to relate modules to clinical traits. We applied the WGCNA R package^63^ on 491,174 CpGs to identify modules significantly associated with AS/PH traits. First, an unsigned co-methylation network was constructed by using *blockwiseModules* function (soft thresholding power = 6, merge cut height = 0.25, and minimum module size = 30). 41 modules were identified from the WGCNA network. DNAm levels of CpGs within a module were summarized by the module eigengene (ME) value which represents the overall methylation level of CpGs clustering in a module. Next, the linear regression model adjusted for covariates was performed between ME value and AS/PH traits for each module to identify significantly associated modules with AS/PH traits. The covariates were the same as EWAS analysis. We considered the module with association p-value < 0.05 as a significant module. Finally, we used CpGs in the significant modules to carry out gene set enrichment analysis.

### Gene set enrichment analysis (GSEA)

We conducted both Gene Ontology (GO) and Kyoto Encyclopedia of Genes and Genomes (KEGG) pathway analysis for each AS/PH trait on two sets of CpGs respectively. The first set of CpGs, the significant EWAS set, was composed of CpGs from the union of both suggestive DMPs and DMRs identified based on suggestive significance of EWAS for each trait of interest. The second set of CpGs, the significant WGCNA-module set, contained CpGs in trait-associated significant modules (P < 0.05) from the WGCNA network. We used the *gsameth* function in the missMethyl^64^ R package that was developed for genome-wide DNAm data to conduct GSEA. The FDR at 5% was applied to GSEA results to correct for multiple testing comparisons.

### Expression quantitative trait methylation (eQTM) analysis

To identify genes whose expression is associated with significant DNAm differences, we conducted eQTM analysis for both suggestive DMPs and DMRs with their annotated genes (Illumina reference table was used to annotate genes for CpGs and DMR annotation was done by coMethyDMR). We used 587 multi-ancestry samples with both MESA Exam 1 RNA-seq normalized gene expression and DNA methylation for association analysis. First, we removed confounding effects in DNA methylation by fitting the linear regression model M value ~ age + gender + race + first 4 genomic PCs of ancestry + estimated cell type proportions (Lymph, Mono, and Neu) and extracting DNA methylation residuals from the model. We used the median M value for the DMR. Similarly, we removed potential confounding effects in RNA-seq by fitting model normalized gene expression ~ age + gender + race + first 4 genomic PCs of ancestry + PEER factors 1–10 and extracting gene expression residuals from the model. Next, for each gene expression and significant DNAm difference pair, we tested association between gene expression residuals (outcome) and DNA methylation residuals via a simple linear regression to quantify eQTM. The FDR at 5% was applied to eQTM results for suggestive DMPs and DMRs respectively to correct for multiple testing comparisons.

### Functional enrichment analysis

To understand the complex interpretation of significant DNAm differences better, we applied eFORGE^65,66^ to test whether our AS/PH trait-associated DNAm differences were enriched in regulatory elements from the Roadmap Epigenomics Consortium^67^ across 20 tissues and cell types. The CpGs included in both suggestive DMPs and DMRs for each AS/PH trait were used for eFORGE 15 chromatin states enrichment analysis. eFORGE selects a background of 1,000 random CpGs with matching properties based on gene-centric categories (first Exon, 3′ untranslated region or UTR, 5′UTR, Body, intergenic region or IGR, TSS1500 and TSS200) and CpG island-centric categories (CpG island, CpG island shore/shelf, N/A or “open sea”). Then the eFORGE uses a 1 kb proximity to filter out highly correlated input CpGs. Finally, eFORGE compares the number of CpGs overlapping regulatory elements from the reference panel with those obtained randomly to calculate enrichment scores for each of the selected cell types. eFORGE performs the Benjamini–Yekutieli approach to account for multiple testing corrections for a cell-type level significance.

### Tissue correlation look-up in GTEx

We used GTEx v8 gene expression data^68^ of both Whole-Blood and Aorta tissue (AS/PH-relevant tissue) to check their tissue correlation for the prioritized genes after follow-up analyses. The GTEx v8 gene expression data was downloaded from the GTEx portal. The inverse normalization was first applied to 360 tissue-overlapped GTEx samples. Then both Pearson’s correlation and its p-value were reported to measure tissue-correlation level.

### Supplementary Information

Supplementary Information 1.

Supplementary Information 2.

## Data Availability

The arterial stiffness and pulsatile hemodynamics data for the Multi-Ethnic Study of Atherosclerosis (MESA) are available by application through dbGaP. The dbGaP accession number for MESA is phs000209. The Infinium Methylation EPIC BeadChip (850 K) array data for MESA/TOPMed participants is available through dbGaP via accession phs001416.v3.p1.
