# Supplementary material for: Multi-ancestry epigenome-wide analyses identify methylated sites associated with aortic augmentation index in TOPMed MESA
Source: Sci Rep. 2023 Oct 17;13:17680. doi: 10.1038/s41598-023-44806-z (PMC10582077; doi:10.1038/s41598-023-44806-z)
Supplement: Supplementary file 1 — Supplementary Information 1. [file 41598_2023_44806_MOESM1_ESM.docx]

**Supplementary Information for “****Multi-ancestry epigenome-wide analyses identify methylated sites associated with aortic augmentation index in TOPMed MESA”**

Xiaowei Hu, Jeongok G. Logan, Younghoon Kwon, Joao A.C. Lima, David R. Jacobs, Daniel Duprez, Lyndia Brumback, Kent D. Taylor, Peter Durda, W. Craig Johnson, Elaine Cornell, Xiuqing Guo, Yongmei Liu, Russell P. Tracy, Thomas W. Blackwell, George Papanicolaou, Gary F. Mitchell, Stephen S. Rich, Jerome I. Rotter, David J. Van Den Berg, Julio A. Chirinos, Timothy M. Hughes, Francine E. Garrett-Bakelman, Ani Manichaikul


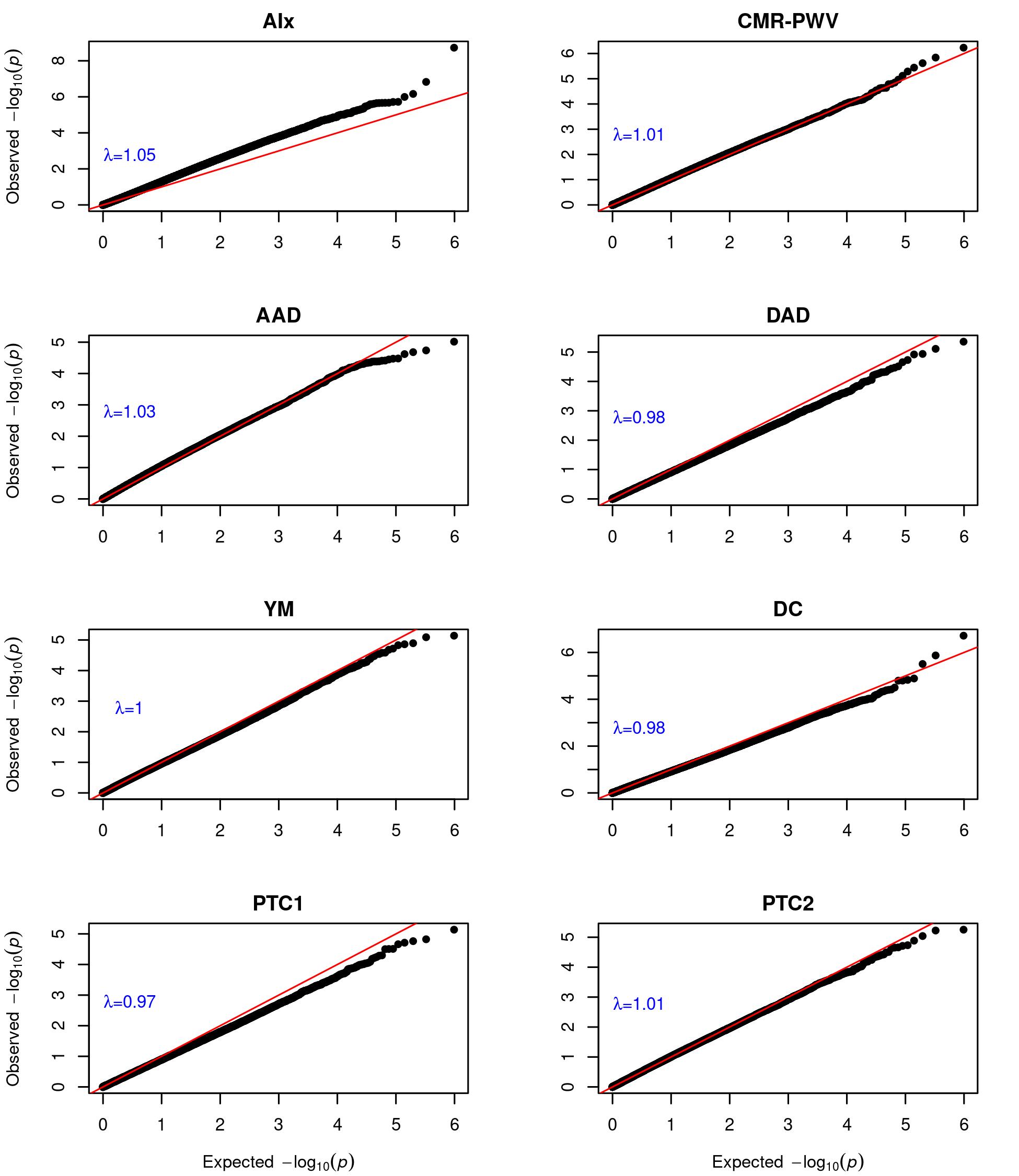


**Supplementary Figure 1:** Quantile-Quantile (QQ) plots of p-values from epigenome-wide association studies between 491,174 CpGs and arterial stiffness and pulsatile hemodynamics traits. AIx, aortic augmentation index; CMR-PWV, aortic arch pulse-wave velocity measured by cardia magnetic resonance imaging; AAD, ascending aortic distensibility; DAD, descending aortic distensibility; YM, Young’s Elastic Modulus; DC, distensibility coefficient; PTC1 and PTC2, radial artery pressure waveform index 1 and 2; __, genomic inflation value.


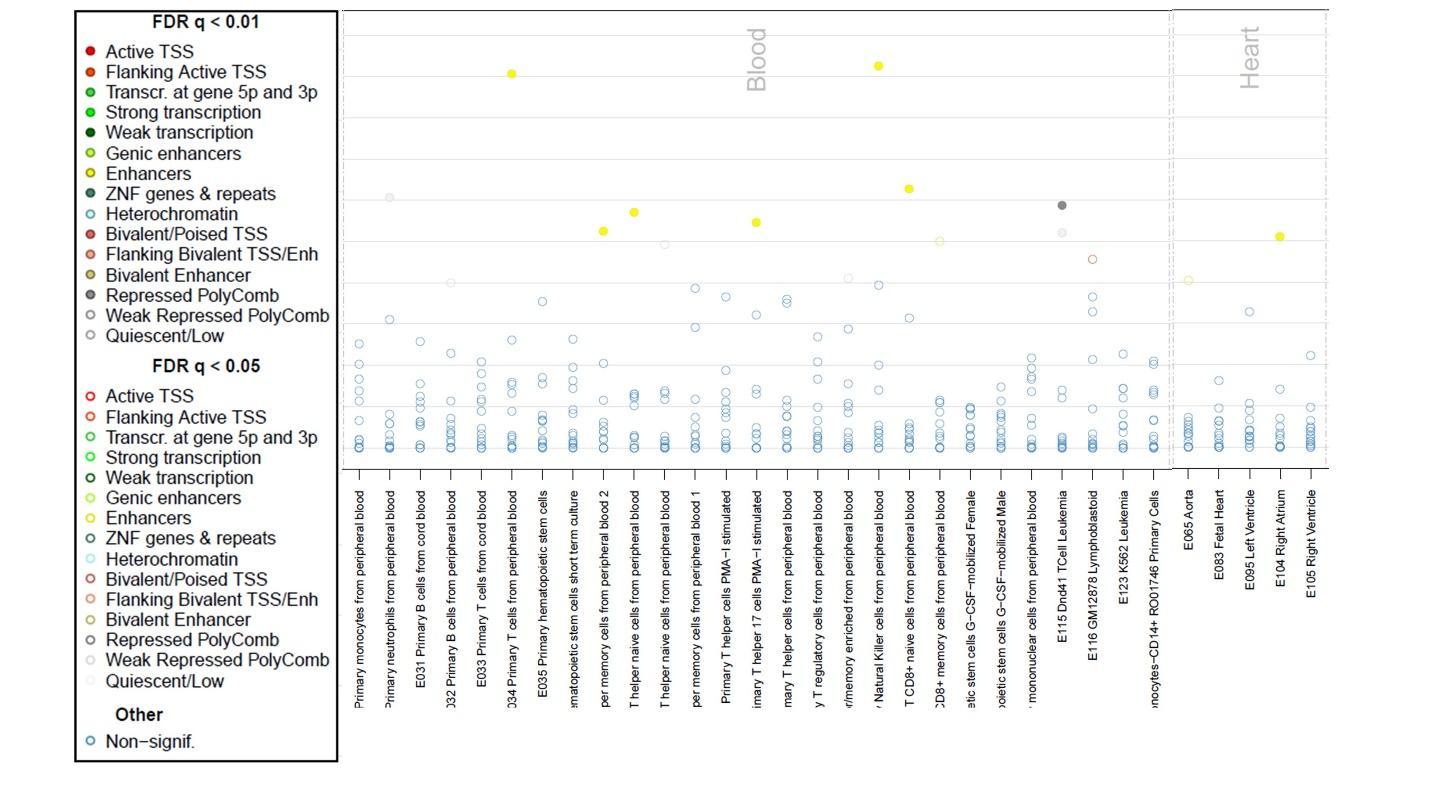


**Supplementary Figure 2:** 15 chromatin states enrichment in blood and heart tissues for CpGs included in AIx-associated differentially methylated positions and regions.


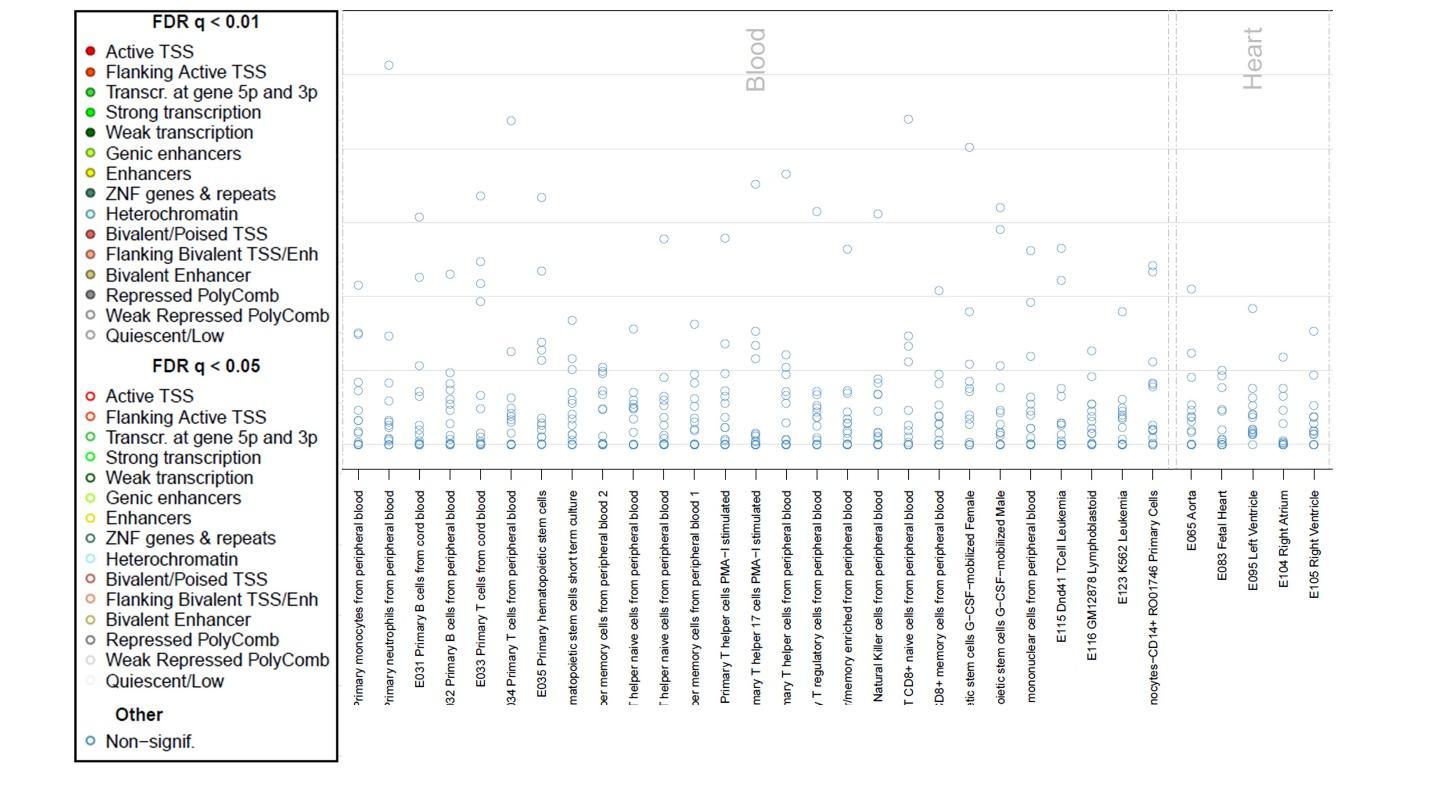


**Supplementary Figure 3:** 15 chromatin states enrichment in blood and heart tissues for CpGs included in CMR-PWV-associated differentially methylated positions and regions.


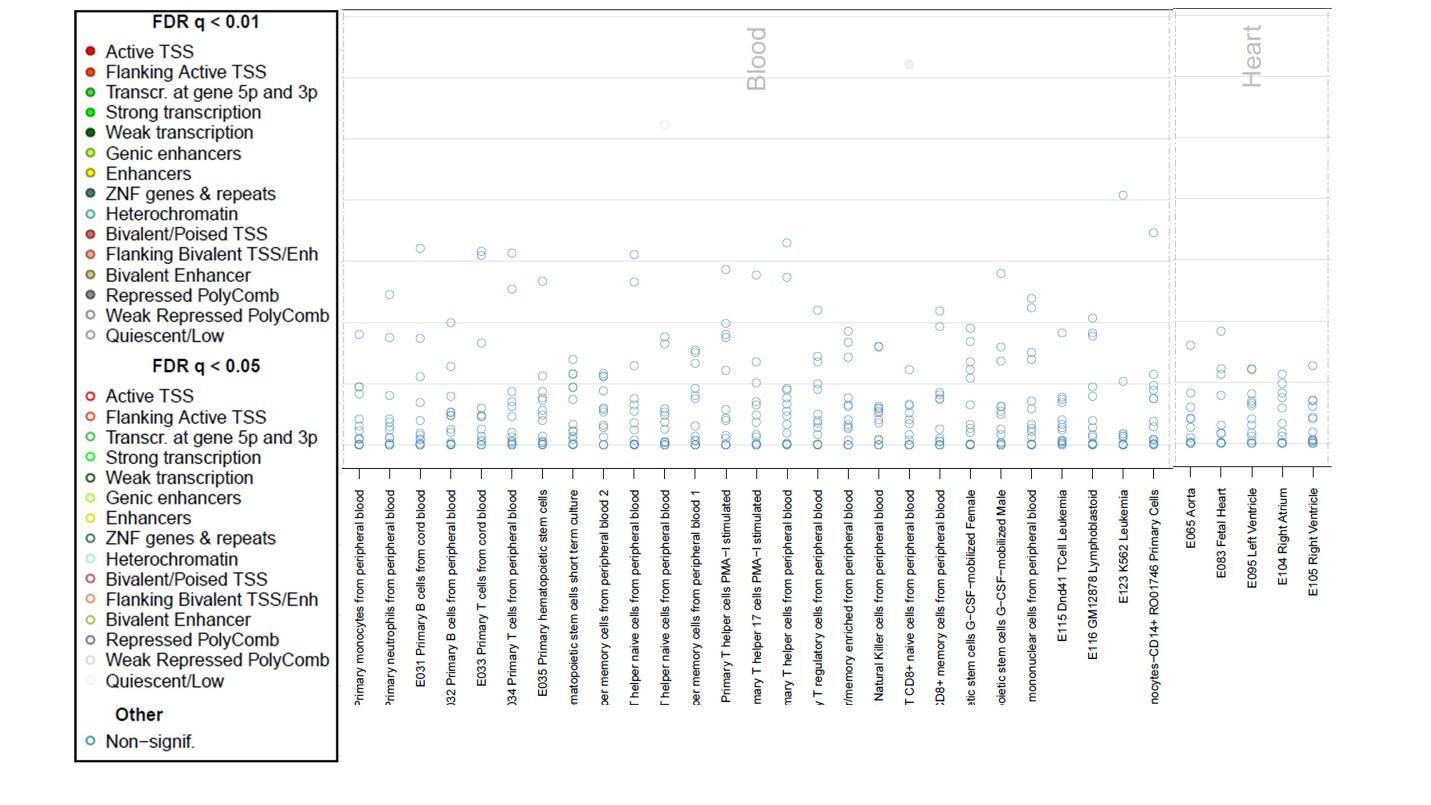


**Supplementary Figure 4:** 15 chromatin states enrichment in blood and heart tissues for CpGs included in AAD-associated differentially methylated positions and regions.


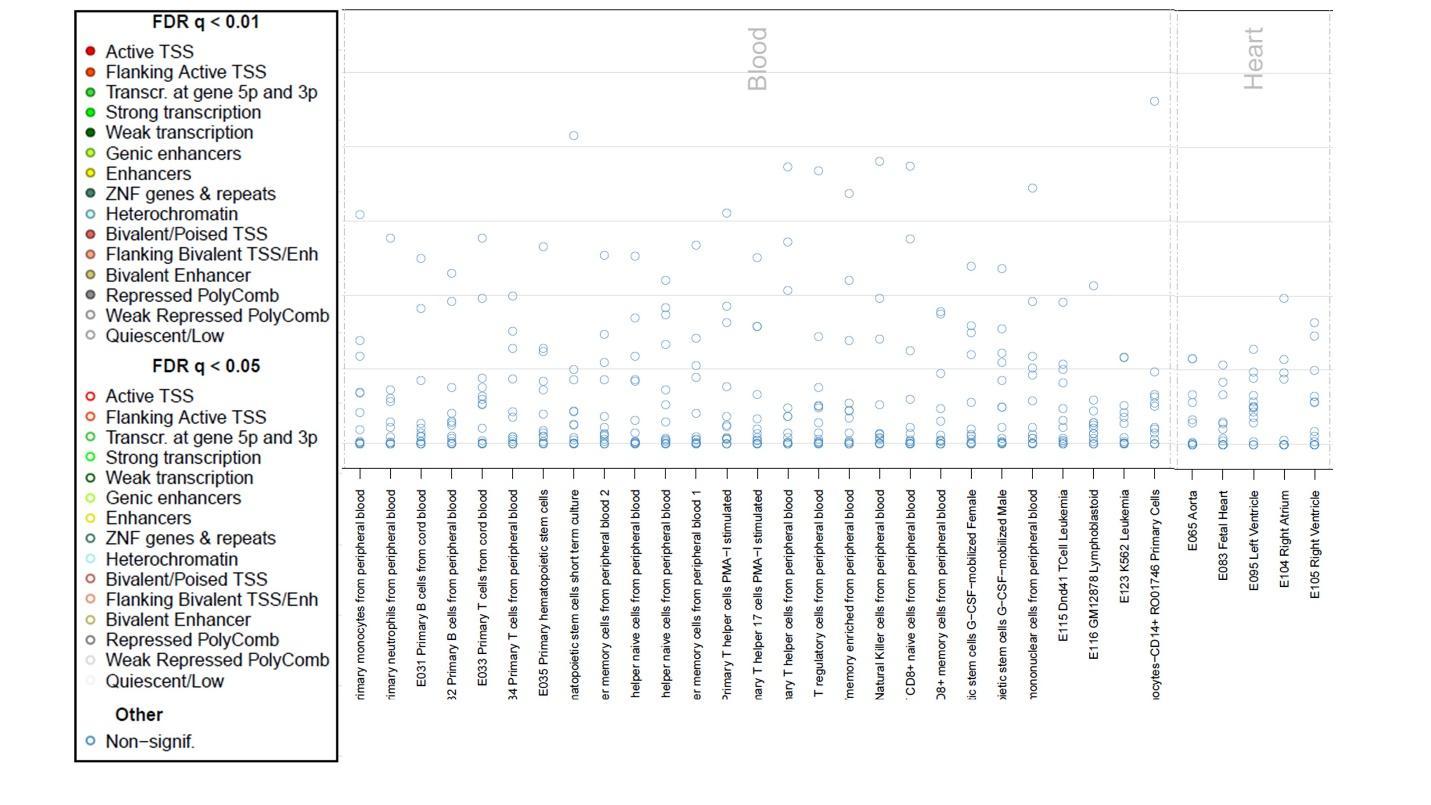


**Supplementary Figure 5:** 15 chromatin states enrichment in blood and heart tissues for CpGs included in DAD-associated differentially methylated positions and regions.


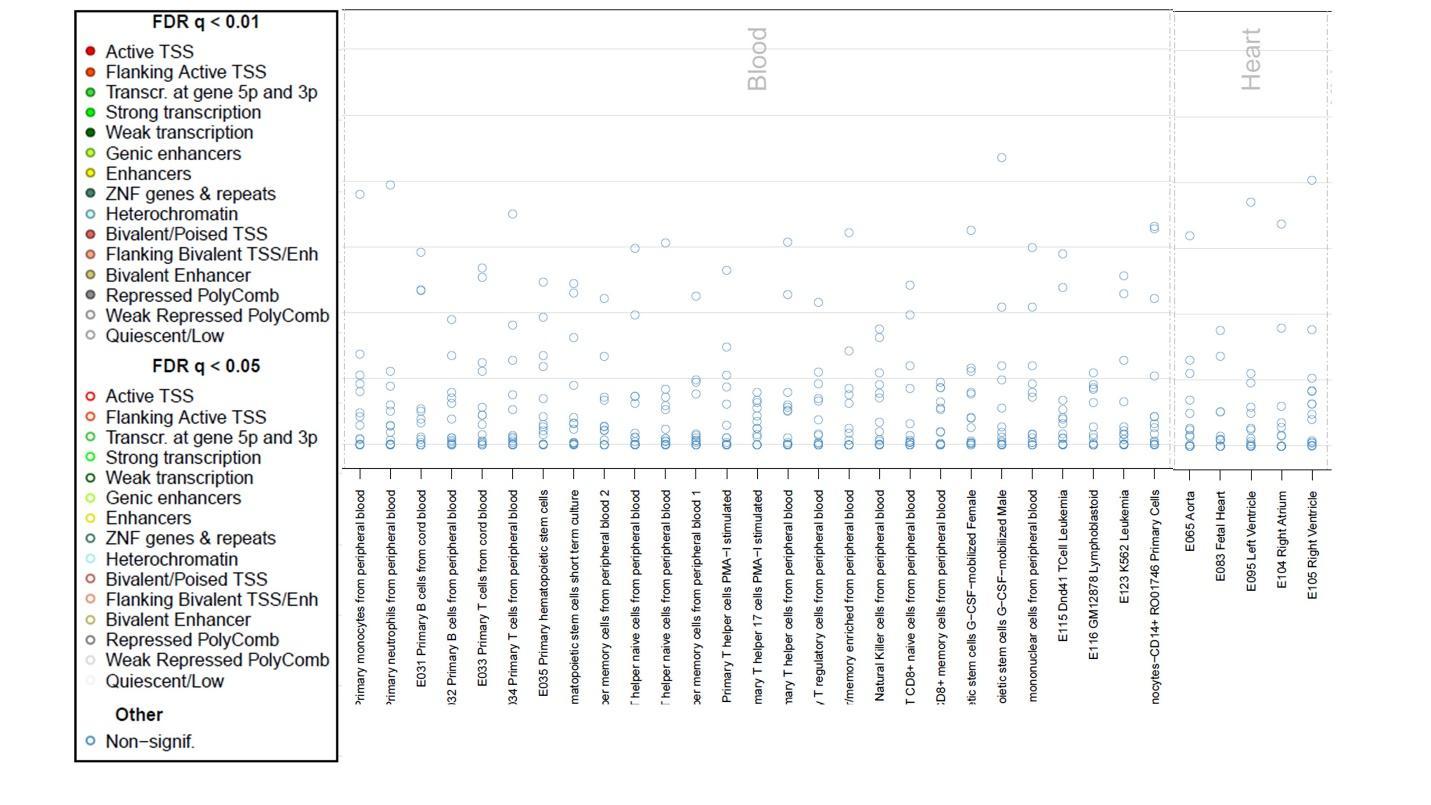


**Supplementary Figure 6:** 15 chromatin states enrichment in blood and heart tissues for CpGs included in YM-associated differentially methylated positions and regions.


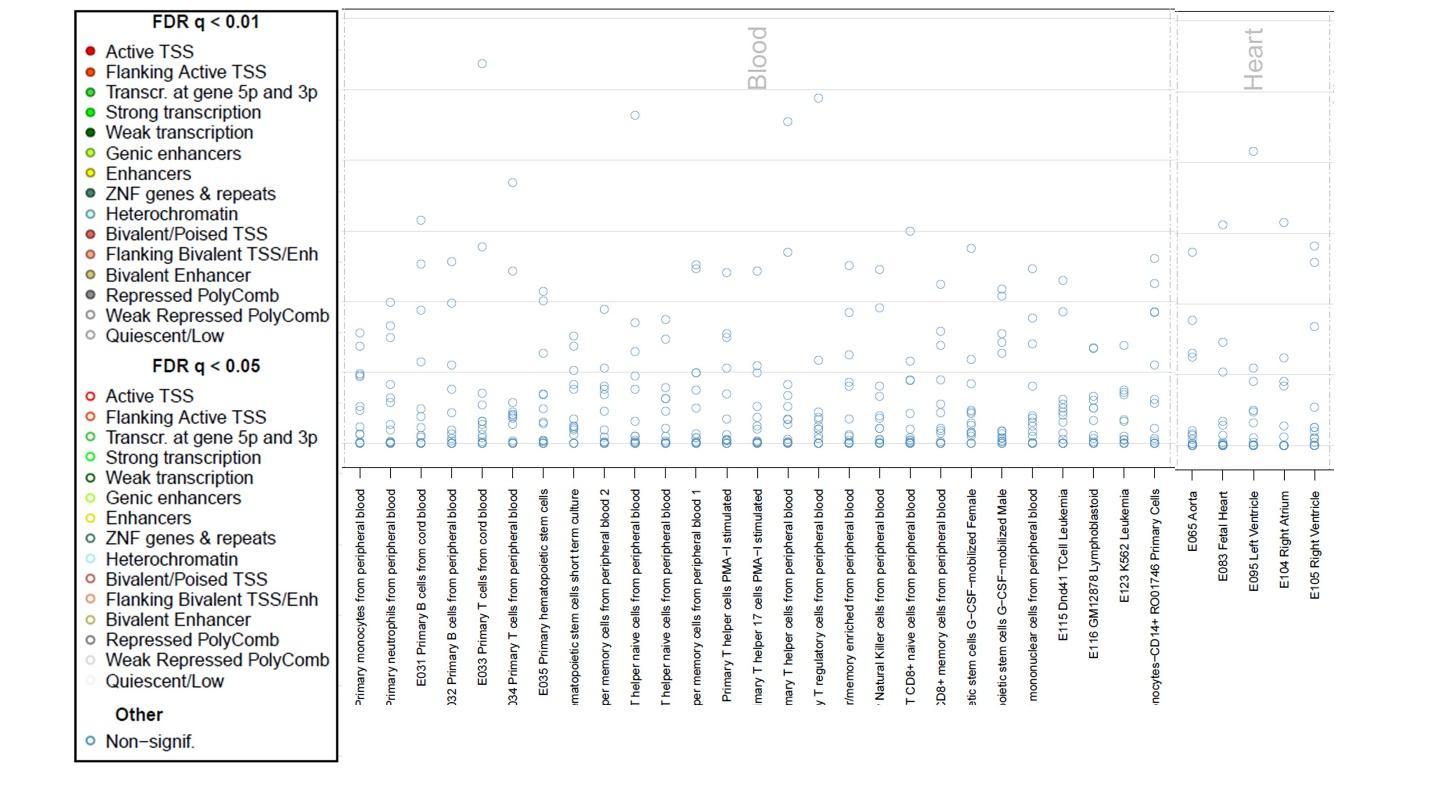


**Supplementary Figure 7:** 15 chromatin states enrichment in blood and heart tissues for CpGs included in DC-associated differentially methylated positions and regions.


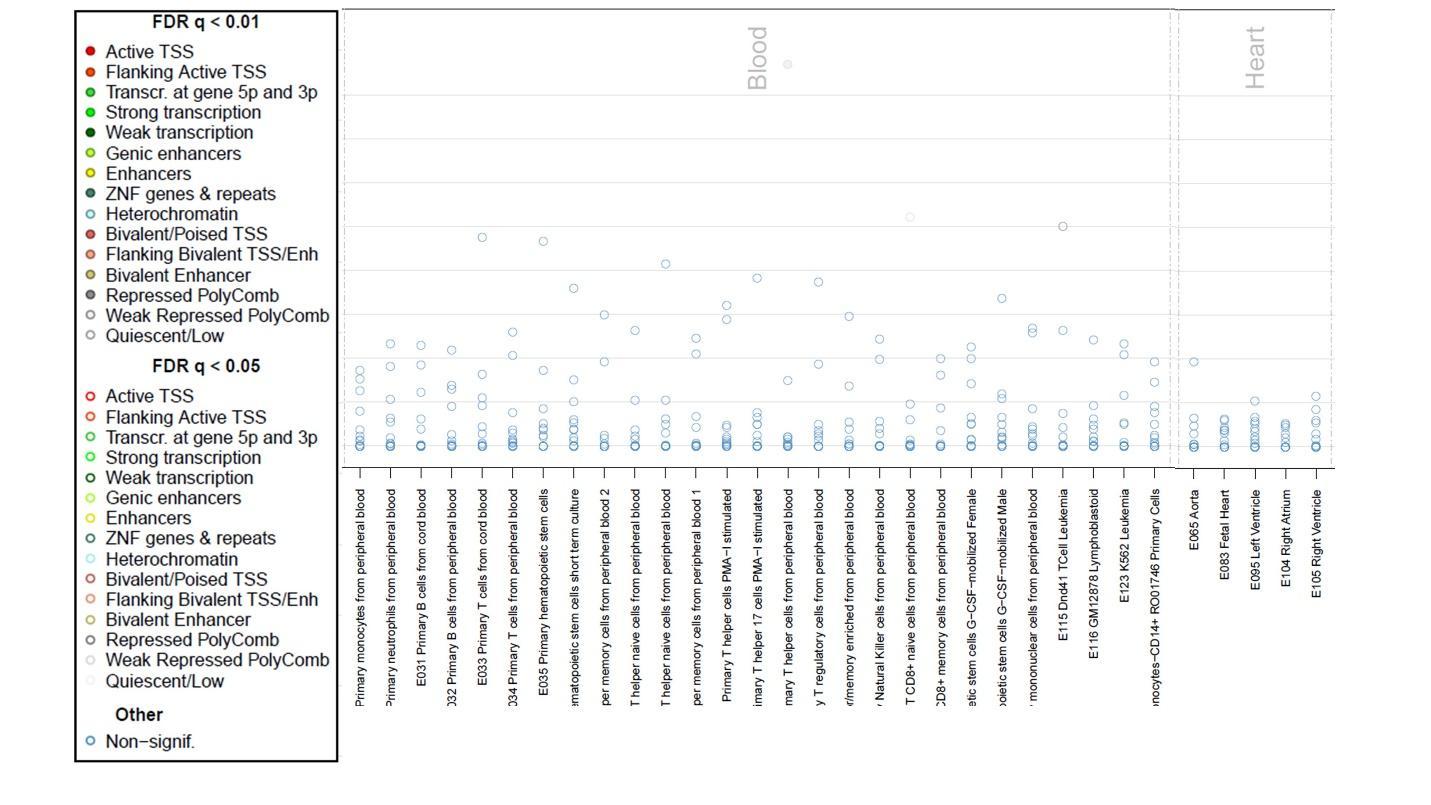


**Supplementary Figure 8:** 15 chromatin states enrichment in blood and heart tissues for CpGs included in PTC1-associated differentially methylated positions and regions.

**
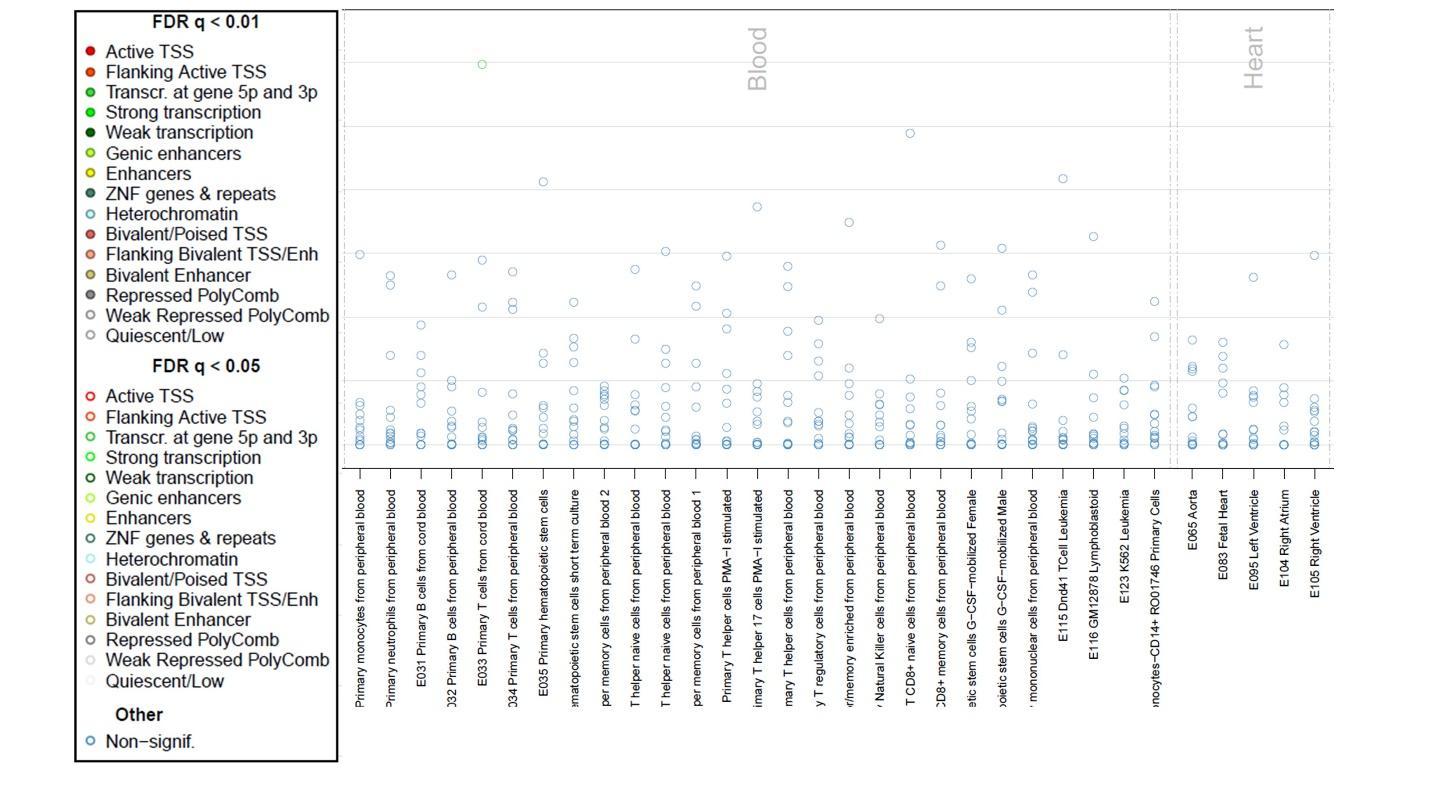
**

**Supplementary Figure 9:** 15 chromatin states enrichment in blood and heart tissues for CpGs included in PTC2-associated differentially methylated positions and regions.

In this section we will include Supplementary Table 2, Supplementary Table 12, Supplementary Table 20, and Supplementary Table 29. The Excel file will contain Supplementary Table 1, Supplementary Table 3, Supplementary Tables 4-11, Supplementary Tables 13-19, Supplementary Tables 21-28 and Supplementary Tables 30-37.

| Phenotype | Epigenome-wide  FDR0.05 | Epigenome-wide  FDR0.1 | Epigenome-wide  P<1e-4 | Candidate-gene  FDR0.05 | Candidate-gene  FDR0.1 | Candidate-gene  P<0.05 | Total  DMPs |
| --- | --- | --- | --- | --- | --- | --- | --- |
| AIx | 2 | 15 | 325 | 0 | 0 | 136 | 461 |
| CMR-PWV | 0 | 0 | 51 | 0 | 0 | 80 | 131 |
| AAD | 0 | 0 | 46 | 0 | 0 | 72 | 118 |
| DAD | 0 | 0 | 22 | 0 | 0 | 51 | 73 |
| YM | 0 | 0 | 35 | 0 | 0 | 57 | 92 |
| DC | 0 | 1 | 22 | 0 | 0 | 56 | 78 |
| PTC1 | 0 | 0 | 19 | 0 | 0 | 42 | 61 |
| PTC2 | 0 | 0 | 31 | 0 | 0 | 70 | 101 |

**Supplementary Table 2:** Number of suggestive differentially methylated positions from epigenome-wide association analysis in MESA. AIx, aortic augmentation index; CMR-PWV, aortic arch pulse-wave velocity measured by cardia magnetic resonance imaging; AAD, ascending aortic distensibility; DAD, descending aortic distensibility; YM, Young’s Elastic Modulus; DC, distensibility coefficient; PTC1 and PTC2, radial artery pressure waveform index 1 and 2; Epigenome-wide FDR, false discovery rate based on multiple testing correction on 491,174 CpGs; Candidate-gene FDR, false discovery rate based on multiple testing correction on 1,289 CpGs that were annotated to 59 candidate genes.

| Phenotype | Epigenome-wide FDR0.05 | Epigenome-wide FDR0.1 | Epigenome-wide  P<5e-4 | Candidate-gene  FDR0.05 | Candidate-gene  FDR0.1 | Candidate-gene  P<0.05 | Total  DMRs |
| --- | --- | --- | --- | --- | --- | --- | --- |
| AIx | 0 | 0 | 13 | 0 | 0 | 2 | 15 |
| CMR-PWV | 0 | 0 | 3 | 0 | 0 | 1 | 4 |
| AAD | 0 | 0 | 0 | 0 | 0 | 0 | 0 |
| DAD | 0 | 0 | 3 | 0 | 0 | 3 | 6 |
| YM | 0 | 0 | 2 | 0 | 0 | 0 | 2 |
| DC | 0 | 0 | 0 | 0 | 0 | 0 | 0 |
| PTC1 | 0 | 0 | 2 | 0 | 0 | 2 | 4 |
| PTC2 | 0 | 0 | 2 | 0 | 0 | 2 | 4 |

**Supplementary Table 12:** Number of suggestive differentially methylated regions from epigenome-wide association analysis in MESA. AIx, aortic augmentation index; CMR-PWV, aortic arch pulse-wave velocity measured by cardia magnetic resonance imaging; AAD, ascending aortic distensibility; DAD, descending aortic distensibility; YM, Young’s Elastic Modulus; DC, distensibility coefficient; PTC1 and PTC2, radial artery pressure waveform index 1 and 2; Epigenome-wide FDR, false discovery rate based on multiple testing correction on 8,165 co-methylated genomic regions; Candidate-gene FDR, false discovery rate based on multiple testing correction on 26 co-methylated genomic regions that were annotated to 59 candidate genes.

| Trait | No. CpGs | No. FDR GO | No. FDR KEGG |
| --- | --- | --- | --- |
| AIx | 518 | 22 | 36 |
| CMR-PWV | 143 | 46 | 14 |
| AAD | 118 | 99 | 47 |
| DAD | 93 | 38 | 9 |
| YM | 98 | 17 | 7 |
| DC | 78 | 82 | 42 |
| PTC1 | 71 | 15 | 5 |
| PTC2 | 115 | 268 | 43 |

**Supplementary Table 20:** Summary of FDR-significant (FDR<0.05) gene set enrichment analysis using CpGs included in the significant EWAS set. AIx, aortic augmentation index; CMR-PWV, aortic arch pulse-wave velocity measured by cardia magnetic resonance imaging; AAD, ascending aortic distensibility; DAD, descending aortic distensibility; YM, Young’s Elastic Modulus; DC, distensibility coefficient; PTC1 and PTC2, radial artery pressure waveform index 1 and 2; No. CpGs, number of CpGs included in the enrichment analysis; No. FDR GO, number of FDR-significant Gene Ontology terms based on multiple testing correction on 22,582 terms; No. FDR KEGG, number of FDR-significant Kyoto Encyclopedia of Genes and Genomes pathways based on multiple testing correction on 347 pathways.

| Trait | Module | No. CpGs | Estimate (se) | P-value | No. FDR GO | No. FDR KEGG | No. Overlap FDR GO | No. Overlap FDR KEGG |
| --- | --- | --- | --- | --- | --- | --- | --- | --- |
| AIx | turquoise | 138,350 | 0.51 (0.20) | 0.0105 | 448 | 48 | 7 | 11 |
|  | grey60 | 1,036 | 0.35 (0.14) | 0.0160 | 0 | 0 | 0 | 0 |
|  | purple | 5,146 | 0.39 (0.16) | 0.0164 | 95 | 1 | 1 | 0 |
|  | saddlebrown | 57 | -0.31 (0.13) | 0.0198 | 0 | 0 | 0 | 0 |
|  | darkorange | 95 | 0.26 (0.13) | 0.0388 | 0 | 0 | 0 | 0 |
| CMR-PWV | yellowgreen | 37 | -1.62 (0.61) | 0.0081 | 0 | 24 | 0 | 0 |
|  | violet | 51 | 1.79 (0.71) | 0.0115 | 0 | 0 | 0 | 0 |
| AAD | blue | 63,303 | -2.34 (1.18) | 0.0488 | 400 | 57 | 4 | 4 |
| DAD | darkgreen | 431 | -2.68 (1.12) | 0.0166 | 0 | 0 | 0 | 0 |
|  | orangered4 | 33 | 2.15 (0.96) | 0.0246 | 0 | 0 | 0 | 0 |
|  | darkorange | 95 | -2.12 (0.96) | 0.0272 | 0 | 0 | 0 | 0 |
| YM | violet | 51 | -1.16 (0.48) | 0.0160 | 0 | 0 | 0 | 0 |
|  | mediumpurple3 | 32 | 0.92 (0.41) | 0.0239 | 75 | 24 | 0 | 0 |
|  | skyblue3 | 36 | -0.88 (0.41) | 0.0309 | 88 | 24 | 0 | 0 |
| DC | darkgrey | 208 | 0.06 (0.02) | 6.95E-04 | 0 | 0 | 0 | 0 |
|  | lightyellow | 798 | 0.03 (0.01) | 0.0010 | 17 | 1 | 1 | 0 |
|  | skyblue3 | 36 | 0.02 (0.01) | 0.0181 | 88 | 24 | 4 | 6 |
|  | violet | 51 | 0.02 (0.01) | 0.0392 | 0 | 0 | 0 | 0 |

**Supplementary Table 29:** Nominal-significant (P<0.05) modules from Weighted Gene Correlation Network Analysis. AIx, aortic augmentation index; CMR-PWV, aortic arch pulse-wave velocity measured by cardia magnetic resonance imaging; AAD, ascending aortic distensibility; DAD, descending aortic distensibility; YM, Young’s Elastic Modulus; DC, distensibility coefficient; PTC1 and PTC2, radial artery pressure waveform index 1 and 2; No. CpGs, number of CpGs included in the module and enrichment analysis; Estimate (se) and P-value, summary statistics from association test between trait and eigen-CpG from module in a linear model adjusted for the same covariates as epigenome-wide association studies; No. FDR GO, number of FDR-significant (FDR<0.05) Gene Ontology terms based on multiple testing correction on 22,582 terms; No. FDR KEGG, number of FDR-significant (FDR<0.05) Kyoto Encyclopedia of Genes and Genomes pathways based on multiple testing correction on 347 pathways; No. Overlap FDR GO/KEGG, number of overlapping FDR-significant Gene Ontology terms/ Kyoto Encyclopedia of Genes and Genomes pathways between significant enrichment results from using two sets of CpGs.
